# Editorial: “Changing the Perspective of the Noble Gas Reactivity”

**DOI:** 10.3389/fchem.2021.658318

**Published:** 2021-03-26

**Authors:** Sudip Pan, Gabriel Merino, Pratim K. Chattaraj

**Affiliations:** ^1^ Institute of Advanced Synthesis, School of Chemistry and Molecular Engineering, Jiangsu National Synergetic Innovation Center for Advanced Materials, Nanjing Tech University, Nanjing, China; ^2^ Fachbereich Chemie, Philipps-Universitt Marburg, Hans-Meerwein-Straße, Marburg, Germany; ^3^ Departamento de Física Aplicada, Centro de Investigación y de Estudios Avanzados, Unidad Mérida, Mérida, Mexico; ^4^ Department of Chemistry, Indian Institute of Technology Kharagpur, Kharagpur, India; ^5^ Department of Chemistry, Indian Institute of Technology Bombay, Mumbai, India

**Keywords:** Reactivity, Noble gas, Fullerene (C_60_), Bonding, Structure

Noble gas (Ng) is undoubtedly a lazy element in the periodic table to show any kind of chemical reactivity toward other chemical entities because of its completely filled valence electronic shell, and large ionic potential and low electron affinity. Thanks to the recent advancements both in the experimental and theoretical domains, it is now known that one can force them to work with the proper chemical environment, and so a noble gas is no longer very “noble” (Grochala, 2007; Khriachtchev et al., 2009; Brock and Schrobilgen, 2013; Pan et al., 2014; Pan et al., 2019; Saha et al., 2019; Jalife et al., 2020). Since with an increase in the size of Ng atoms, the outer electronic shell is more loosely bound by nucleus, and therefore the heavier Ng atoms have better aptitude to take part in bonding with other elements. The compounds of Kr, Xe, and Rn are well-known, albeit to a smaller number for the latter case because of the associated radioactivity. The first Ar compound, HArF was only isolated in 2000 in a low-temperature Ar matrix (Khriachtchev et al., 2000). Ne was reported to form very weak complexes with highly electrophilic centers as in NeAuF, NeBeS, NeBeCO_3_, NeBeSO_2_, (Ne)_2_Be_2_O_2_, (NeAr)Be_2_O_2_, and (NeKr)Be_2_O_2_ (Zhang et al., 2014; Yu et al., 2016; Zhang et al., 2017). In a remarkable study, recently Dong et al. synthesized solid compound of helium and sodium Na_2_He at a high pressure (Dong et al., 2017). Therefore, presently all the members of Ng group are known to form chemical bonds. The aim of the present research topic is to highlight the present status of the noble gas chemistry to the readers as well as to report new molecules of Ng and the study of bonding therein. This collection includes nine articles involving 48 authors, among them three are minireviews and six are original articles.

The minireview by Grandinetti summarizes the contributions made in the cationic noble gas hydrides, which are relevant in outer space (Grandinetti, 2020). The author beautifully shows the structure, stability and mode of formation of different such species that range from simple NgH^+^ diatomic molecule to (H_3_^+^)(Ng)_n_. In a comprehensive review, Sanloup elaborates how high temperature and high pressure in planetary interiors induce interesting reactivity in Ng atoms (Sanloup, 2020). This review shows different kind of cage compounds, stoichiometric oxides and metals, and non-stoichiometric compounds having Ng atoms (mostly Xe and in some cases He) which are formed in planetary interiors. Xe was found to take part in a different kind of bonding, however, helium does not take part in bonding. Another review by Miao plays the same tone that Ng can display a nice variety of chemistry under pressure (Miao, 2020). He highlights the types of chemical roles and interactions that Ng exhibits under high pressure, including their oxidizing and reducing properties, Ng-Ng bond formation, aerogen bonding, and reliever of repulsive electrostatic interactions. In an elegant perspective article, Warneke and co-workers elaborated their contributions where anionic systems act as superb electrophiles to bind Ng atoms (Rohdenburg et al., 2020).

Zhang et al. in their study on the bonding in HXeY (Y = Cl, Br, I) and HXeY HX (X = OH, Cl, Br, I, CN, CCH) introduced another view on the H-Xe bond (Zhang et al., 2020). They argued that the H-Xe bond in HXeY is not a classical covalent bond rather a charge-shift bond. In their contribution, Gomila and Frontera showed the systems having “noble gas bond” where an Ng center acts as a Lewis acid (Gomila and Frontera, 2020). On the other hand, Ghara and Chattaraj theoretically proposed viable Ng-Au complexes where frustrated Lewis pair is also involved (Ghara and Chattaraj, 2020). Special emphasis is made on the related bonding situation. In a couple of contributions, Liu and co-workers (Li et al., 2020), and Sarkar and co-workers (Paul et al., 2020) studied confinement effects on the bonding and reactivity of Ng_2_ inside fullerenes.

As guest editors, we would like to thank all the contributing authors, particularly for their work in this pandemic time. We hope that this collection of noble gas chemistry will provide an excellent account of the present state-of-the-art in this field.
